# Dr. Prichard on Physical and Animal Life

**Published:** 1830-04-01

**Authors:** 


### A REVIEW OF THE DOCTRINE OF A VITAL PRINCIPLE, AS MAINTAINED BY SOME WRITERS ON PHYSIOLOGY. WITH OBSERVATIONS ON THE CAUSES OF PHYSICAL AND ANIMAL LIFE. By *J. C. Prichard*, M.D. F.R.S. &c.

DR. PRICHARD has been long and favourably known to the public and to the profession by his great and first work on the varieties of the human race and subsequently by strictly professional publications. The present performance is equally interesting to the general and professional reader; but the difficulty of the subject will probably render it less satisfactory than either of Dr. Prichard's former lucubrations. We are unable to afford space in this journal for a general review of the work, and shall content ourselves with an analysis of a couple of sections—the fifth and sixth—entitled "*a Review of the Controversy respecting the Existence of an Immaterial Principle or Soul—Metaphysical Argument.*"

Our ingenious author observes that the reasons adduced by philosophers in favour of the soul's existence, may be classed under two heads—metaphysical and inferential argument. The *former* consists, not in any attempt at demonstration, but of reasons derived from the nature of the human faculties and modes of thought—the *latter*, in argument by induction. The metaphysical argument is thus stated by Dr. Prichard.

"It has often been observed, that the whole sum of human knowledge may be divided into two departments, with reference to the channels through which it is acquired. The ideas, which belong to one of these departments, are originally acquired by perception, and through the medium of our external senses; those of the other class are revealed to us by our internal consciousness. The objects which are made known to us by the former, are the properties and affections of body, or matter; those which are shown to us by the latter, are the operations of mind. It is self-evident, that all properties whatever, must, as metaphysicians say, *inhere in some substratum*, or, in other words, they must be the qualities of some essence or substance; they must belong to something. The essence or entity to which the first set of properties belong, is, by common agreement, termed body or matter; that to which the second class belong, is named soul, spirit, mind.

"It is a very important remark in this place, and in the argument which is to be stated, that of the two entities or substances now mentioned, matter and spirit, we have no knowledge whatever, except what is merely relative. We only know that such things exist, by inferring that they must exist, because their properties or attributes are perceived or felt. The essential nature of matter is utterly unknown, and the word means nothing at all, except that unknown substance to which certain known properties belong. Of mind or spirit we are equally ignorant, and the words mean nothing at all, but the unknown essence or substance to which another set of known properties belong. Algebraical symbols, such as $x$ and $y$, would be just as expressive as these words, if there were only a general consent to use them for the same purpose. Those persons who have not been accustomed to examine accurately the limits of their knowledge, will not be prepared to admit that their ignorance is equal in these two cases. The remarks I have just made will, therefore, require some further illustrations.

"In the one case, indeed, I apprehend that there will be no hesitation or difference of opinion. All will be disposed readily to admit that we are quite ignorant of the essential nature of mind. Its properties or phenomena are made known to us by our consciousness, or internal feeling. These phenomena include all our intellectual operations, as perception, memory, judgment, or ratiocination, imagination, and the rest; all our emotions, grief, joy, pleasure, pain, passion; all the active propensities, or rather their conscious operation, belong to this part of our nature,—

> "Quidquid agunt homines, votum, timor, ira, voluptas,
> "Gaudia.

"These phenomena are perceived by us with the greatest degree of certainty, but we have no information respecting the nature or manner of subsistence of that part of our being by which they are witnessed, and of which they are affections. We term it, from some of its known qualities, intellect, mind or *mens*, meaning that which knows or understands." 40.

The less reflecting part of the community think they have a more distinct notion of matter, inasmuch as they handle and feel it, and appear to themselves to perceive its immediate contact with their hands and bodies. We cannot follow our learned author in his arguments to prove that we have, in reality, no correct or distinct notion of the real nature of matter. These arguments will be found between page 40 and page 47 of the work under review.

"If the preceding observations are allowed to be well-founded, we shall now be enabled to state, in a plain and intelligible manner, the controversy between the materialists, who deny the real existence of mind or spirit, and those philosophers who maintain it. The proposition set up by the materialist is, that these unknown substances, which I have termed $x$ and $y$, are in reality but one and the same thing, though displaying two different sets of properties or qualities. This is denied by their opponents, who maintain that there are two distinct natures or entities, which, though sometimes brought into union, are essentially distinct from each other.

"1. The advocates for this latter opinion, who are termed Immaterialists, or Spiritualists, remark, in the first place, that there is nothing, in the mode in which we become acquainted with these two classes of phenomena, that tends to favour the supposition that they belong to the same substance. On the contrary, we cannot contemplate them through the medium of the same faculty. One set of phenomena are only known to us, as I have already said, through the medium of our consciousness, or internal feeling; the other are objects of sensation and external perception.

"2. If we attempt to compare these two classes of phenomena with each other, in order to determine whether they are of the same kind, and may probably belong to the same entity, we cannot discover the most remote analogy between them. On the contrary, there results from the contemplation of the one class, a notion which is quite irreconcilable with a notion that results from the contemplation of the other class. We cannot subject matter and its attributes to our examination, without conceiving *it*, as well as the space which it occupies, to be in its nature infinitely divisible, or capable of separation and division without end. On the other hand, we cannot contemplate the phenomena of mind, or make the mental operations of which we are conscious the subjects of our reflection, without being irresistibly convinced that they belong to one being or thing, which is in itself absolutely indivisible. Our perceptions, recollections, thoughts, feelings, emotions of sorrow or joy, are all felt by us to belong to a self-same being, which we cannot, by the utmost effort of imagination, fancy to be divided into parts, or separated. We feel and know it to be a monad, a single and indivisible being. This is the only instance in which the qualities of one of these natures, or essences, can enter into any sort of relation with those of the other, and here they come into the relation of contrast.

"These considerations are strongly in favour of the opinion that $x$, and $y$, the entity to which the properties of matter, and that to which those of mind belong, are distinct in nature. On the other hand, an attempt has been made, by a distinguished natural philosopher and a most subtle metaphysician, who may be considered as the greatest advocate of materialism in modern times, to prove that there is but one kind of substance or being in existence. The following is a short statement of his argument. We never witness the phenomena of mind, except in connexion with those of matter; therefore, the substance to which these two classes of phenomena belong, is one and the same. This is a clear and intelligible proposition, and it deserves a careful examination. If the assertion contained in it were established in its fullest extent, I do not perceive how, in the present state of our knowledge, we could avoid the inference. I shall cite the words in which this inference is drawn by Dr. Priestley, that I may not incur any risk of an erroneous statement.

" 'The power of sensation,' he says, 'or of perception, never having been found *but* in conjunction with a certain organized system of matter, (he alludes to the brain and nervous system,) we ought, as philosophers, to conclude that this power necessarily exists in and results from that organized system, unless it can be shewn to be incompatible with other known properties of the same substance.'

"This proposition is expressed in terms too limited for the argument with which it is connected; but, if we may be allowed to put it into a more general form, it will amount to the following assertion. Mental phenomena are witnessed by us, only in connexion with organized systems of matter; or, to speak plainly, we nowhere discover the manifestations of mind, thought, consciousness, intelligence, except in connexion with brains. Unless the proposition has this meaning, it has no force whatever in the argument; and, if it is received in this general sense, it holds out to the Spiritualist a challenge, which he may very safely accept. He may observe that the whole universe displays the most striking marks of the existence and operation of mind or intellect, in a state separate from organization, and under conditions which preclude all reference to organization. 'The universal Mind,' says a distinguished philosopher,* 'though everywhere present, where matter exists, though everywhere moving and arranging the parts of matter, appears to do so without being united with matter, as in the case of visible, created beings.' There is, therefore, at least one being or substance of that nature which we call mind, separate from organized body." 52.

This consideration, Dr. P. thinks, affords a sufficient reply to Dr. Priestley's argument.

"If the phenomena of mind can be discovered in one instance, in a state absolutely separate from organized matter, it is philosophical to conclude, when we find these phenomena connected and organized, with the properties of which they have nothing in common, that the connexion is accidental, or owing to some particular and temporary circumstances—and that it is not natural and essential."

In the sixth section of Dr. Prichard's work, the learned author takes up the second argument of those philosophers who contend that man is not *merely* a body, but a compound being, uniting a soul and a body. This second argument is inferential. It is an attempt to prove that the phenomena of mind cannot, in conformity with the results of universal experience, be derived from the properties of matter, whether organized or, in any imaginable way, modified.

" 'In all combinations† whatever of material parts, it is found that the powers or properties of the entire system, are nothing more than the sum or aggregate of the powers or properties of the component parts. Figure, magnitude, and motion, (in which last are included attraction and repulsion,) are the universal powers or properties of material particles; and, from the combinations of material particles nothing has ever been known to result, but some modification of figure, magnitude, and motion.' It is, therefore, directly in opposition to the results of universal experience, to suppose that a combination of material particles can give rise to the phenomena, or constitute the operations peculiar to

* "The late Professor Dugal Stewart."
† Belsham's Essays.

mind, or those feelings of which we are internally conscious. The contractions and lengthenings of cords or fibres, and the movements of fluid particles, will ever be something different in kind from the emotions of pleasure and pain, hope and joy.

"This is a very brief and summary statement of a proposition, which it would require some space of time fully to develop and explain. Some persons may, at first, be inclined to question whether it is true, in fact, that in all combinations of material parts, the properties of the whole are nothing more than a modification or aggregate of properties belonging to the parts. It may be observed that, in chemical combinations, the compound body has often different chemical properties from those which belonged to the substances of which it was compounded; and that pieces of machinery display mechanical powers, which could not have been looked for in their parts when separate. Powers which do not come under either of these denominations, are likewise found to result from a combination of materials which, separately, possessed nothing analogous to them. An Eolian harp, composed of pieces of wood and strings, emits musical sounds; and an electrical or galvanic apparatus displays its peculiar properties, which nobody could discover in single jars of glass, or plates of metal. The reply to these objections is as follows. In the first place, with respect to chemical compounds, it is remarked, that all the properties of chemical agents resolve themselves into modifications of one property, *viz.* that of chemical affinity, or the generic or elective attractions of particles of various kinds for each other. This one property, from which all the operations of chemistry result, is modified, and, with respect to particular substances, increased or diminished by chemical composition, but still nothing new in kind is acquired, or can be imagined to be acquired, by any chemical combination whatever. In like manner, the mechanical powers, which are the results of machinery or construction, may be explained, without proving that any power new in kind has been generated. The operations of which the entire system is rendered capable, are nothing more than the obvious result of the joint operations of all the parts, and it is brought about by the compounded agency of their weight, density, elasticity, and other previously existing powers or properties, combined and set into action under particular circumstances. In the instance again of the Eolian harp, or that of the electrical machine, though these instruments are compounded of materials which, by themselves, possessed neither musical nor electric properties, still nothing new is produced by the fabrication, because a new agent is in reality called into play. In the one case, the air in motion or wind, in the other, the electric fluid, displays by means of the machine its well-known properties, and produces, by its peculiar movements, in the one instance, an agitation of the atmosphere upon our ears, in the other, an electric shock. On the whole, it is, perhaps, not too much to conclude it to be an universal fact, that the properties possessed by any aggregates whatever of material bodies, are not different in kind from the properties of the parts from which such aggregates were compounded, but are all resolvable into them. The inference which philosophers have drawn from these premises, in proof of the existence of an immaterial principle of feeling and intellect, is sufficiently obvious. The human brain, which, in a certain way, is instrumental in the operations of the mind, can only be an inferior organ or auxiliary, assisting the mind in the performance of its functions, as a crutch or a pair of spectacles assists a man in walking or seeing. Mental phenomena, as thought, feeling, volition, cannot be affections of the brain itself, which is an organized structure, composed wholly of insentient and inert particles of matter, because the former are phenomena entirely peculiar; and because powers or qualities, so diverse from those of the component parts, are never, within the sphere of human experience, found to be produced by any combinations.

"To the apparent conclusiveness of this argument it has been objected, by some metaphysicians, that 'the Creator, being omnipotent, *could* endow material particles, in a state of organization, with whatever powers it pleased him to bestow.' This is granted by the advocates of an immaterial principle, but then they make the following comment upon it. If the powers bestowed upon any system of parts were such as did not result from any possible modification or composition of the powers of the parts, the endowing such a system with new powers or properties, *can, apparently*, mean nothing *else* than the adding to it of some new thing—some new entity possessed of such properties, or to the nature of which they have some relation; and this is, in fact, the very supposition with which the Spiritualist or Immaterialist sets out, *viz.* that an immaterial principle of mind or intellect is superadded to the organized structure of the brain.

"I have thus endeavoured to give a summary of the arguments by which philosophers have endeavoured to establish the existence of a separate, sentient, percipient, or cogitative principle, distinct from the brain and nervous system, as well as from the whole organic structure of the body, but by the constitution of things capable at present of coming into relations with the external world only through the instrumentality of the bodily organs. How far these arguments are conclusive, I leave it to others to determine." 59.

We do not expect that these arguments will prove conclusive among the determined materialists. The question is evidently insusceptible of proof either way. The Spiritualist cannot demonstrate the existence of a soul—the Materialist cannot disprove its existence. There is a consciousness within the human breast, almost universal, that man has something beyond the material organs of which he appears to be composed—and this consciousness is strengthened, if not confirmed by revelation. This is all that need be said on the business in the present stage of our existence.

Wait the great teacher Death, and God adore.

